# Effect of Cryotherapy and Occlusal Reduction on Postoperative Endodontic Pain in Mandibular First Molars with Symptomatic Apical Periodontitis: A Prospective, Parallel, Double-Blinded Randomized Controlled Trial

**DOI:** 10.1055/s-0044-1791219

**Published:** 2024-11-07

**Authors:** Lana Almasoud, Tarek Elsewify, Ranya Elemam, Bassem Eid

**Affiliations:** 1 Department of Restorative Dental Sciences, College of Dentistry, Gulf Medical University, Ajman, United Arab Emirates; 2 Department of Endodontic, Faculty of Dentistry, Ain Shams University, Cairo, Egypt

**Keywords:** cryotherapy, occlusal reduction, postoperative pain, symptomatic apical periodontitis, symptomatic irreversible pulpitis

## Abstract

**Objectives** This study aimed to compare the intensity of postoperative pain following the final rinse using cold saline compared with room temperature saline and occlusal reduction.

**Materials and Methods** A prospective, parallel, double-blinded randomized controlled trial was conducted on 69 first mandibular molars diagnosed with symptomatic irreversible pulpitis and symptomatic apical periodontitis. Single-visit root canal treatment was performed; access cavity preparations and chemomechanical preparations were carried out using the ProTaper Gold rotary system under copious irrigation using 3% sodium hypochlorite and 17% EDTA. The patients were divided into three groups ( *n* = 23): control group: room temperature saline (25°C) final rinse without occlusal reduction; cryotherapy group: cold saline (2.5–4°C) final rinse without occlusal reduction; and occlusal reduction group: room temperature saline with occlusal reduction. Pain scores were recorded using the visual analog scale preoperatively and postoperatively via telephone at 6, 24, 48, 72 hours, and 7 days intervals. Age data were analyzed using one-way analysis of variance followed by Tukey's post hoc test. Pain score data were analyzed using Kruskal–Wallis' test followed by Dunn's post hoc test for intergroup comparisons and Friedman's test followed by Nemenyi's post hoc test for intragroup comparisons. Correlations were analyzed using Spearman's rank-order correlation coefficient. The significance level was set at *p* < 0.05 within all tests.

**Results** Cryotherapy reduced postoperative pain compared with the control group with a statistically significant difference at 24 hours only ( *p* = 0.016). At other intervals, no statistically significant difference in pain score was measured between all three groups ( *p* > 0.05). After 7 days, all patients recorded a zero pain score.

**Conclusion** Cryotherapy was as effective as the occlusal reduction in reducing postoperative pain in cases of symptomatic irreversible pulpitis with symptomatic apical periodontitis, significantly more than the control group.

## Introduction

Postoperative pain is a common sequela after root canal treatment and is triggered by periapical inflammation. This inflammation may be present as either symptomatic or asymptomatic. 1 Mechanical elements, such as overinstrumentation, may contribute to postoperative pain as it promotes the enlargement of the apical foramen. Excessive pressure or overinstrumentation during treatment may result in debris extrusion, in which microorganisms and infected or irritating materials are pushed past the apex of the root, producing postoperative discomfort and inflammation. Chemical factors also play a role, since some irrigating solutions and medicaments used during the root canal treatment can irritate the surrounding tissues if pushed beyond the apex and not thoroughly rinsed out. 2 3 4

Understanding these etiologies is crucial in managing and minimizing postoperative pain for patients undergoing root canal treatment. Several strategies have been presented in endodontics for managing postoperative pain such as using long-lasting anesthesia 5 and prescribing analgesics such as nonsteroidal anti-inflammatory drugs, acetaminophen, and corticosteroids. 6 7 8 Although they are relatively safe medications, they have been linked to gastrointestinal intolerance, as well as renal, hepatic, and respiratory disorders. 9 10

Occlusal reduction has been implemented as a method for postoperative pain management reducing the risks of systemic analgesics prescription. 11 Occlusal reduction is the selective grinding of the occlusal surface of the tooth. This approach minimizes the force applied when tooth height and occlusal contacts are reduced decreasing mechanical stimulation of the sensitized nociceptors. Various studies have been performed to evaluate the effects of occlusal reduction on the treatment of postoperative pain and discomfort. 12 13 14 However, whether it is an effective intervention in the management of postoperative pain after root canal therapy remains controversial. 15 16

Cryotherapy is another relatively novel and conservative method of pain control that has gained attention in the last decade as a viable and conservative adjuvant for the management of postoperative pain. 17 Cryotherapy is a treatment in which a particular body segment is exposed to cold temperatures. It aims to decrease the targeted tissue temperature to promote healing and aid in other therapeutic effects such as diminishing edema, inflammation, and pain. 17 Cryotherapy used in dentistry has decreased pain after periodontal surgeries, extractions, and implant surgeries. In 2015, intracanal cryotherapy was introduced in the endodontic field as a successful treatment for reducing postoperative endodontic pain. 17 One way of utilizing cryotherapy in endodontics is by applying it as a final cold irrigating solution into the canal. 18 Several studies demonstrated a significant reduction in postoperative pain after cryotherapy. 19 20 21

To date, no one has compared the effects of cryotherapy application with occlusal reduction in managing postoperative pain. There is a lack of sufficient evidence to support or reject the use of cryotherapy as a routine solution in cases of symptomatic apical periodontitis. Therefore, this study evaluated the effects of cryotherapy applications on postoperative pain on root canal-treated mandibular first molars with symptomatic apical periodontitis in comparison to occlusal reduction. The null hypothesis assumes no difference in the intensity of postoperative pain between the room temperature saline, cryotherapy, and occlusal reduction in patients with symptomatic irreversible pulpitis and symptomatic apical periodontitis.

## Materials and Methods

### Study Design, Ethical Approval, and Trial Registration

**Fig. 1:**
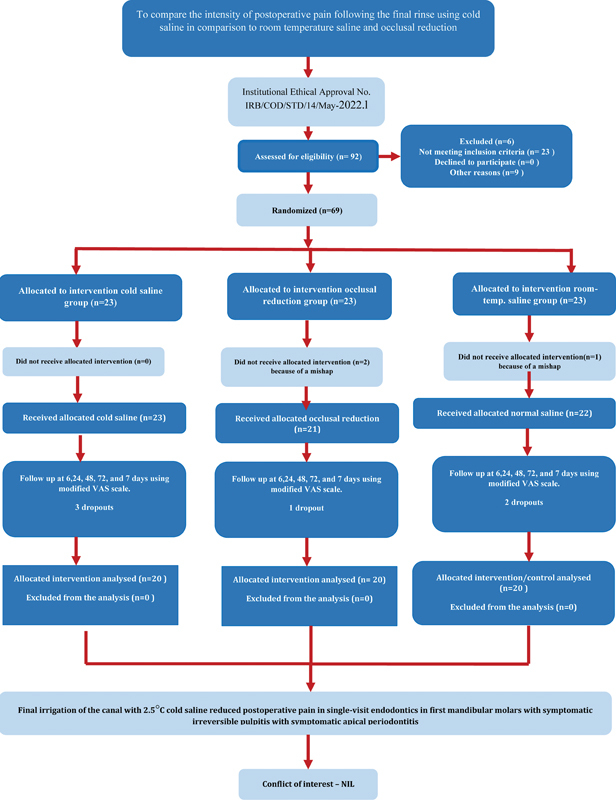
PRIRATE flow diagram for reporting randomized trials in endodontics.

The study was designed as a prospective, parallel, double-blind randomized clinical trial. The study was approved by the local Institutional Review Board with reference number IRB/COD/STD/14/May 2022. This clinical trial has been registered at http://www.ClinicalTrials.gov with the number NCT05722704. Reporting of this study was done following PRIRATE 2020 guidelines for reporting randomized trials in endodontics as demonstrated in Fig. 1 .

### Sample Size Calculation

A power analysis using IBM SPSS statistics (version 27) was developed to have sufficient power to apply a statistical test of the null hypothesis that no difference between tested groups would be detected. Using an alpha error of 0.05, power of 0.8, and an effect size of 1.02, the estimated sample size calculated based on a previous study by Sylvia Western et al 22 was 60 patients with 20 first mandibular molars in each group ( *n* = 20) and increased to 69 ( *n* = 23) to compensate for the 15% dropout.

### Sample Selection

Patients requiring root canal treatment at the MDS Endodontics postgraduate clinics, starting from September 2022 to December 2023, meeting the eligibility criteria listed below were enrolled after receiving a thorough explanation of the procedure, including its risks and other treatment options, and signing an informed consent.

The study included participants aged between 18 and 70 years who possess permanent mature mandibular first molars diagnosed with symptomatic irreversible pulpitis and symptomatic apical periodontitis. Only healthy patients without systemic diseases were considered, with normal occlusal contact a prerequisite. Additionally, the study required participants to comply with the outlined protocols and procedures.

The exclusion criteria for this study encompassed pregnant patients, individuals with mental disorders, and those with a history of long-term corticosteroid use. Additionally, participants who had experienced intraoperative complications were excluded from the study.

### Randomization and Blinding

The patients were randomly allocated into three groups ( *n* = 23) according to the ratio of 1:1:1. Randomization was performed by using computer-generated randomization software ( www.random.org ).

*Allocation concealment* : Before the trial, random sequence generation, allocation concealment, and intervention preparation were completed. To prevent selection bias during the recruitment phase, the third author ensured that the allocation sequence was withheld from the operator (first author) by sealing it in an opaque envelope.

*Implementation* : Before the endodontic treatment, the third author communicated the patient's assigned group and the designated final irrigating solution to the operator (first author). As a result, participants in each of the three study groups remained unaware of both the final rinse utilized and their respective group assignments. Furthermore, to mitigate assessment bias, all pain assessment forms and data were overseen and collected by a trained clinician (second author) who was also kept unaware of the study details.

### Patient Management

The patients were inquired about their chief complaint and the reason for seeking treatment. A detailed medical history was obtained followed by the dental history and chronology of events that led to his/her chief complaint.

Extraoral examination and intraoral examination were performed to reach a definitive diagnosis. The vitality was assessed using a thermal cold stimulus with a cotton pellet damped with Endo-Ice refrigerant (Coltene, Ohio, United States) for 5 seconds and electric pulp testing, D640 Digitest II Pulp Vitality Tester (Parkell, Ohio, United States). Mobility, palpation, and percussion tests were done to assess periapical health. An intraoral periapical radiograph was also performed.

Enrolled patients were inquired about the intensity of pain pretreatment and requested to fill in their visual analog scale (VAS) after a thorough description of the scale by the assessor. The VAS is represented by a horizontal line of 100 mm (10 cm) with one end indicating no pain and the other indicating worst pain (score 10). There are five categories of pain intensity: no pain (level 0), mild pain (levels 1–3), moderate pain (levels 4–6), severe pain (levels 7–9), and worst pain (level 10).

### Endodontics Treatment Procedure

A single operator, the first author, performed the treatment on all the patients in a single visit. All the patients received one cartridge of local anesthesia 2% lidocaine with 1:100,000 epinephrine (Septodont, Paris, France). Each tooth was isolated using a rubber dam sheet. The access cavity was prepared using a round bur number 4 (Johnson-Promident, New York, United States) and tapered fissure diamond bur number 173 (Johnson-Promident) under copious water using a high-speed handpiece.

After the removal of coronal pulp tissues using a sterile excavator, the working length was determined with #10 stainless steel hand K-files (Dentsply Sirona, Ballaigues, Switzerland) with the use of the Root ZX II apex locator (J. Morita, Tokyo, Japan) and confirmed using intraoral periapical radiographs. Hand instrumentation was completed till the #15 K file to create a glide path. All the canals were further prepared using ProTaper Gold rotary files (Dentsply Sirona, Erlangen, Germany) up to file F2 (25/0.08) using the Endo MateTC2 endodontic motor (NSK, Tokyo, Japan) at a speed of 350 rpm and torque of 2.5 N-cm. 2 The molar was filed up to F3 (30/0.09) if it only had one distal canal. Apical patency was maintained throughout the shaping procedure using the #10 K file.

All the canals were irrigated with 10 mL of 3% sodium hypochlorite between each file change during the whole preparation procedure using a 27-gauge side-vented Navi Tip needle (Ultradent Product, Utah, United States) 2 mm away from the working length with the aid of a rubber stopper for 5 minutes. Manual dynamic activation with a fitted master cone was utilized for the last wash of 10 mL of 3% sodium hypochlorite. Further irrigation by 10 mL of saline and 1 mL of 17% EDTA for 1 minute to remove the smear layer was utilized, and manually activated.

In the control group, the final irrigation was completed with 20 mL room temperature saline at 25°C, for 5 minutes using a 27-gauge side-vented needle 2 mm away from the working length. In the cryotherapy group, 20 mL of cryotreated saline maintained at 2.5 to 4°C was irrigated for 5 minutes by a 27-gauge side-vented needle 2 mm away from the working length in each canal as a final rinse. The saline was placed in a refrigerator with a thermometer sensor (Cooper Atkins, Connecticut, United States) to confirm the 2.5 to 4°C temperature range. After the removal, the temperature of the cold saline was preserved by keeping the irrigation syringes in a special box filled with ice and the digital thermometer (Cooper Atkins). In the occlusal reduction group, final irrigation was completed with 20 mL room temperature saline at 25°C, for 5 minutes using a 27-gauge side-vented needle 2 mm away from the working length.

In all groups, the canals were partially dried using paper points (Diadent, Songjeong-dong, Korea) and obturated using the single cone technique, by selecting a fitted ProTaper Gold gutta-percha master cone (Dentsply Sirona) and a Totalfil bioceramic sealer (FKG, Le Crêt-du-Locle, Switzerland). A postobturation intraoral periapical radiograph was performed to evaluate the obturation.

A permanent composite restoration (Ivoclar-Vivadent, Schaan, Liechtenstein) was placed after obturation. In the control and cryotherapy groups, high points were checked by an articulating paper and adjusted using a diamond polishing bur. In the occlusal reduction group, occlusal contacts on the functional and nonfunctional cusps, as well as the marginal ridges, were reduced/ground by 1 mm. 23 The reduction is completed by the use of a diamond bur number 173 in a high-speed handpiece and confirmed with an articulating paper. Intraoral visualization was also done to confirm occlusal space when the patient is in centric occlusion.

### Follow-up

The assessor (second author) contacted all the patients via telephone and inquired about the pain's intensity in the root canal treatment area at various intervals: 6, 24, 48, 72 hours, and 7 days. The patients were requested to answer the call and describe their pain as explained before initiating the treatment. Analgesic pills that had been taken by the patient after treatment were also inquired about and recorded on the patient's VAS.

### Statistical Analysis

Categorical data were presented as frequency and percentage values and were analyzed using the chi-square test. Numerical data were presented as mean, standard deviation, median, and interquartile range values. They were analyzed for normality using Shapiro–Wilk's test. Age data were normally distributed and were analyzed using one-way analysis of variance followed by Tukey's post hoc test. Pain score data were nonparametric and were analyzed using Kruskal–Wallis' test followed by Dunn's post hoc test for intergroup comparisons and Friedman's test followed by Nemenyi's post hoc test for intragroup comparisons. Correlations were analyzed using Spearman's rank-order correlation coefficient. The significance level was set at *p* < 0.05 within all tests. Statistical analysis was performed with R statistical analysis software version 4.3.2 for Windows.

## Results

Intergroup comparisons and summary statistics for demographic data are presented in Table 1 and Supplementary Material Appendix 1 (available in the online version only). There was no significant difference between the groups regarding gender and age ( *p* > 0.05). Seven cases in the control group and two cases in the cryotherapy and occlusal reduction groups took medication, and the difference between the groups was not statistically significant ( *p* = 0.062) ( Table 2 ).

**Table 1:** Intergroup comparisons and summary statistics for demographic data

Parameter		Control	Cryotherapy	Occlusal reduction	Test statistic	*p* -Value
Gender, *n* (%)	Male	11 (55.00%)	17 (85.00%)	12 (60.00%)	4.65	0.098 a
	Female	9 (45.00%)	3 (15.00%)	8 (40.00%)		
Age (y)	Mean ± SD	34.00 ± 7.85	31.40 ± 6.56	32.35 ± 8.98	0.56	0.574 a

Abbreviation: SD, standard deviation.

Note: Significant ( *p* < 0.05); nonsignificant ( *p* > 0.05).

a Nonsignificant.

**Table 2:** Intergroup comparisons, frequencies, and percentages for medication intake

Medication intake	Control, *n* (%)	Cryotherapy, *n* (%)	Occlusal reduction, *n* (%)	Test statistic	*p* -Value
No	13 (65.00%)	18 (90.00%)	18 (90.00%)	5.57	0.062 a
Yes	7 (35.00%)	2 (10.00%)	2 (10.00%)		

Note: Significant ( *p* < 0.05); nonsignificant ( *p* > 0.05).

a Nonsignificant.

After 24 hours, the control group had a statistically significantly higher pain score than the cryotherapy group ( *p* = 0.016), as represented in Table 3 . At other intervals, no statistically significant difference in pain score was measured between all three groups ( *p* > 0.05). After 7 days, all patients recorded a zero pain score.

**Table 3:** Intergroup comparisons, mean ± SD in addition to median and IQR values for pain score at all intervals

Time	Measures	Pain score			Test statistic	*p* -Value
		Control	Cryotherapy	Occlusal reduction		
Preoperative	Mean ± SD	7.60 ± 2.60 ^A^	7.65 ± 2.16 ^A^	7.85 ± 1.98 ^A^	0.07	0.968 a
	Median (IQR)	8.50 (3.25) ^A^	8.00 (3.25) ^A^	8.00 (3.00) ^A^		
6 h	Mean ± SD	3.65 ± 3.03 ^A^	1.85 ± 1.81 ^A^	2.15 ± 2.11 ^A^	4.27	0.118 a
	Median (IQR)	3.00 (4.50) ^A^	2.00 (3.00) ^A^	2.00 (4.00) ^A^		
24 h	Mean ± SD	2.20 ± 1.82 ^A^	0.70 ± 1.13 ^B^	1.20 ± 1.32 ^AB^	8.32	0.016 b
	Median (IQR)	2.00 (3.25) ^A^	0.00 (2.00) ^B^	0.50 (2.25) ^AB^		
48 h	Mean ± SD	0.85 ± 1.76 ^A^	0.25 ± 0.79 ^A^	0.50 ± 0.95 ^A^	2.98	0.225 a
	Median (IQR)	0.00 (1.00) ^A^	0.00 (0.00) ^A^	0.00 (0.25) ^A^		
72 h	Mean ± SD	0.25 ± 0.72 ^A^	0.10 ± 0.45 ^A^	0.05 ± 0.22 ^A^	1.71	0.424 a
	Median (IQR)	0.00 (0.00) ^A^	0.00 (0.00) ^A^	0.00 (0.00) ^A^		
7 d	Mean ± SD	0.00 ± 0.00 ^A^	0.00 ± 0.00 ^A^	0.00 ± 0.00 ^A^	NA	NA
	Median (IQR)	0.00 (0.00) ^A^	0.00 (0.00) ^A^	0.00 (0.00) ^A^		

Abbreviations: IQR, interquartile range; NA, not applicable; SD, standard deviation.

Note: Values with different superscript letters within the same horizontal row are significantly different; significant ( *p* < 0.05); nonsignificant ( *p* > 0.05).

a Nonsignificant.

b Significant.

For all groups, there was a statistically significant difference in pain score values measured at different intervals ( *p* < 0.001). For the control and occlusal reduction groups, post hoc pairwise comparisons showed that the pain score measured preoperatively was significantly higher than that of other intervals ( *p* < 0.001). In addition, they showed values measured after 6 hours to be significantly higher than values measured after 48, 72 hours, and 7 days ( *p* < 0.001). Finally, the results showed values measured after 24 hours to be significantly higher than values measured after 72 hours and 7 days ( *p* < 0.001). For the cryotherapy group, they showed preoperative value to be significantly higher than values measured at other intervals ( *p* < 0.001). In addition, they showed values measured after 6 hours to be significantly higher than values measured at later intervals ( *p* < 0.001) ( Table 4 ).

**Table 4:** Intragroup comparisons, mean ± SD in addition to median and IQR values for pain score at all the intervals

Group	Measure	Pain score						Test statistic	*p* -Value
		Preoperative	6 h	24 h	48 h	72 h	7 d		
Control	Mean ± SD	7.60 ± 2.60 ^A^	3.65 ± 3.03 ^B^	2.20 ± 1.82 ^BC^	0.85 ± 1.76 ^CD^	0.25 ± 0.72 ^D^	0.00 ± 0.00 ^D^	56.48	<0.001 a
	Median (IQR)	8.50 (3.25) ^A^	3.00 (4.50) ^B^	2.00 (3.25) ^BC^	0.00 (1.00) ^CD^	0.00 (0.00) ^D^	0.00 (0.00) ^D^		
Cryotherapy	Mean ± SD	7.65 ± 2.16 ^A^	1.85 ± 1.81 ^B^	0.70 ± 1.13 ^C^	0.25 ± 0.79 ^C^	0.10 ± 0.45 ^C^	0.00 ± 0.00 ^C^	128.80	<0.001 a
	Median (IQR)	8.00 (3.25) ^A^	2.00 (3.00) ^B^	0.00 (2.00) ^C^	0.00 (0.00) ^C^	0.00 (0.00) ^C^	0.00 (0.00) ^C^		
Occlusal reduction	Mean ± SD	7.85 ± 1.98 ^A^	2.15 ± 2.11 ^B^	1.20 ± 1.32 ^BC^	0.50 ± 0.95 ^CD^	0.05 ± 0.22 ^D^	0.00 ± 0.00 ^D^	143.51	<0.001 a
	Median (IQR)	8.00 (3.00) ^A^	2.00 (4.00) ^B^	0.50 (2.25) ^BC^	0.00 (0.25) ^CD^	0.00 (0.00) ^D^	0.00 (0.00) ^D^		

Abbreviations: IQR, interquartile range; NA, not applicable; SD, standard deviation.

Note: Values with different superscript letters within the same horizontal row are significantly different; significant ( *p* < 0.05); nonsignificant ( *p* > 0.05).

a Significant.

## Discussion

Postoperative pain following the completion of a root canal treatment is a common occurrence, often taking place because of the periapical inflammatory response to numerous triggers of a chemical, mechanical, or microbial nature. 3 This inflammatory response presents clinically as symptomatic apical periodontitis and the management of this condition is challenging.

Various factors have been proposed to influence postoperative pain following endodontic treatment, including preoperative pain level, and pulp and periapical status. 24 To ensure standardization of the groups in the study, the inclusion criteria included mandibular first molar teeth diagnosed with symptomatic irreversible pulpitis and symptomatic apical periodontitis. Vital teeth have been known to show a higher incidence and intensity of postoperative pain following root canal treatment when compared with necrotic teeth. 25 26 This inclusion criterion was selected due to the high predictability of postoperative pain as reported in previous studies. 25 26

It has been reported that some medical conditions could decrease the success rate of root canal treatments. Systemic diseases such as osteoporosis, diabetes, and cardiovascular diseases were found to prevent or delay periapical healing following endodontic treatment. 27 To exclude the influence of any systemic health-related factors on the outcome of the endodontic treatment performed, patients with systemic diseases were excluded. Moreover, the root canal procedure was completed in a single visit to reduce the potential of coronal microleakage through temporary restorative material. 28 29

In our study, 92 patients were assessed for eligibility, a total of 69 participants met the eligibility criteria and were randomized. Three participants did not receive the allocated intervention and were excluded from the study due to a procedural mishap during the treatment procedure. Six participants were excluded from the study after receiving the allocated intervention because they did not respond to telephone calls to record the pain scores postoperatively at the allocated time intervals. Consequently, the final sample that has undergone analysis consisted of 60 patients.

The concealment of the randomization sequence was ensured by employing sequentially numbered opaque sealed envelopes to prevent selection bias. Furthermore, double blinding was upheld, ensuring that patients remained unaware of their assigned groups. The treatment for all the participants was completed by a single operator (first author), whereas the follow-up by telephone was achieved by the assessor (second author), unaware if an experimental intervention was administered. Similarly, the preoperative pain score was recorded by the assessor (second author). This precaution was implemented to minimize potential bias in the study results.

The severity of pain was assessed in the literature using different methods such as Wong–Baker faces pain rating scale, Heft-Parker pain scale, and VAS. 20 22 30 In the current study, VAS was used due to its reliable and simple nature, making it easy for patients belonging to different age groups and backgrounds to provide accurate feedback on the intensity of their postoperative pain scores. 31

The preoperative pain score, across all three groups, did not present a statistically significant difference. This uniformity in preoperative pain score is advantageous as it aids in establishing a baseline for comparison. Moreover, this warrants that any observed variations in postoperative pain scores between the groups can be ascribed to the intervention being studied rather than preexisting pain discrepancies. Reducing preoperative pain fluctuations allows the study to more precisely evaluate the intervention's efficacy and develop reliable findings on how it affects pain management.

Several methods for managing postoperative endodontic pain have been previously adopted, including prescribing the appropriate dose of postoperative nonsteroidal anti-inflammatory drugs, 32 or intraoperative techniques such as injecting long-acting anesthesia, 5 occlusal reduction, 12 and the most conservative method which is cryotherapy application. 33 In this study, we have evaluated the effect of reducing postoperative pain in cases diagnosed as symptomatic irreversible pulpitis with apical periodontitis using either cryotherapy or occlusal reduction compared with an active comparator group with room temperature saline final rinse and no occlusal reduction.

Occlusal reduction is an approach for managing postoperative pain following endodontic treatment. Within this study, a reduction of 1 mm was carried out on functional cusps, nonfunctional cusps, and marginal ridges, and subsequently confirmed with an articulating paper, as outlined by Zaman et al. 23 Further confirmation was done by visualizing the space between the opposing tooth when in centric occlusion.

Intracanal cryotherapy, a relatively novel conservative therapeutic approach, involves the administration of extremely low temperatures of a solution into the root canal system to reduce postoperative pain. In the cryotherapy group of the current study, a temperature of 2.5°C saline was employed based on the findings of Vera et al, 17 indicating that a 5-minute application of 2.5°C saline irrigant can effectively reduce the root surface temperature by more than 10°C. The maintenance of the temperature of the cold saline adhered to the methodology outlined in Al-Nahlawi et al's study. 18 This involved placing the saline in a refrigerator with a thermometer and subsequently storing it in a box with ice to ensure temperature control with a thermometer as well. A 27-gauge side-vented needle was used 2 mm short of the working length to deliver the cold saline and ensure temperature reduction throughout the entire root length.

The null hypothesis tested in the current study was partially rejected, as cryotherapy was deemed effective in postoperative pain reduction in cases diagnosed with symptomatic irreversible pulpits with symptomatic apical periodontitis, where a statistically significant difference was found at the 24-hour interval.

The results of this study revealed a statistically significant difference at 24 hours only between the cryotherapy and control groups. The cryotherapy group exhibited the lowest pain score at 24 hours, followed by the occlusal reduction group, and finally, the control group. The effect of intracanal cryotherapy in reducing postoperative pain at 24 hours can be explained by the physiological reaction that takes place, leading to a reduction of postoperative pain, which relies heavily on the process of vasoconstriction. Once vasoconstriction occurs, as well as the subsequent reduction in blood flow and decreased permeability in the area of concern, edema and inflammatory responses are also diminished, which in turn, alleviates pain. In addition, cryotherapy plays a role in modulating nerve conduction in the form of reducing the speed of transmission of pain signals, providing a sensation of pain relief. Moreover, cryotherapy has also been directly linked to the reduction of the release of certain chemical mediators responsible for leading the sensation of pain such as tumor necrosis factor-α and interleukin (IL)-6. 34 35

In agreement with the current study, Shah et al 36 reported a statistically significant difference at the 24-hour interval in postoperative pain following a single-visit endodontic treatment between a group receiving cryotherapy and another receiving normal saline irrigation. Moreover, Keskin et al 37 also revealed significantly lower VAS scores of patients belonging to the cryotherapy group as opposed to those belonging to the control group, at the 24-hour interval. Additionally, Bazaid and Kenawi 38 noted a significant difference in postoperative pain between the cryotherapy and control groups at 24 hours.

In contrast to our findings, Alharthi et al 30 reported no significant difference in their cryotherapy and control groups at 6, 24, and 48 hours. This can be attributed to the difference in inclusion criteria, where their study's sample included only asymptomatic cases. Similarly, Akpinar and Kaya 39 also revealed no significant difference between the cryotherapy and control groups. This, however, can be explained by the different methodologies. The aforementioned study conducted their root canal treatment throughout two visits rather than one, in addition to placing a calcium hydroxide dressing for the 7-day duration between both visits.

Furthermore, Al-Nahlawi et al reported significant reductions in mean pain scores in the cryotherapy group during the 6-hour follow-up. 18 Similarly, in another study by Gundogdu and Arslan, 40 the intracanal cryotherapy group had significantly lower mean pain scores than the control group at the 72-hour follow-up. The discrepancies observed from our study between the 6-hour and 72-hour follow-ups can be attributed to the distinct peak of postendodontic pain that occurs during the 24- to 48-hour follow-up period, which makes it less noticeable during the early (6 hours) and later (72 hours) stages of follow-up. 24

Although not statistically significant, the occlusal reduction group showed lower pain scores than the control group at all intervals. The rationale for the decreased incidence of postoperative pain is due to a biological phenomenon termed mechanical allodynia. This refers to an increased sensitivity to certain mechanical stimuli, such as biting or percussion, as a result of raised levels of mediators responsible for activating the peripheral terminals of nociceptors. Through occlusal reduction, it is possible to bypass this phenomenon by eliminating the mechanical stimuli which could potentially lead to the occurrence of postoperative pain. 41

The literature shows contradicting results about the effect of occlusal reduction compared with placebo or control groups. In support of our results, Vianna et al 42 reported no statistical significance in pain perception after occlusal reduction for teeth with irreversible pulpitis. In addition, Ghimire et al 43 also concluded that occlusal reduction does not affect postoperative pain scores. In contradiction to our results, Emara et al 12 demonstrated a reduction in postoperative pain in posterior mandibular teeth diagnosed with symptomatic pulpitis and apical periodontitis between the control group and the occlusal reduction group. While there exists a discrepancy in postoperative VAS scores between both groups at 6, 12, 24, and 48 hours, the only statistically significant difference was at the 12-hour interval. 23 44

Patients belonging to both intervention groups, either cryotherapy or occlusal reduction, exhibited lower levels of postoperative pain in comparison to the control group at all time periods. There was, however, a lack of a statistically significant difference between the cryotherapy, occlusal reduction, and saline groups at 6, 12, 48, 72 hours, and 7 days. Although the difference was not statistically significant, the cryotherapy group continuously experienced the least postoperative pain followed by occlusal reduction. This suggests that, despite the absence of statistical significance at other intervals, the cryotherapy group consistently felt less pain than the other groups. As the pain scores values fail to demonstrate normal distribution and were nonparametric, we would recommend another randomized controlled trial with a larger sample size, based on our results, which will help elaborate the significance of this difference.

According to the findings of the current study, no postoperative pain was reported on the seventh day. To explain this occurrence, it is important to note that several cytokines, including IL-1β and IL-6, are known to play a role in the development of apical periodontitis. 35 IL-10, on the other hand, is an anti-inflammatory cytokine that plays an important role in the prevention of autoimmune and inflammatory diseases. 45 Emad et al, using polymerase chain reaction, discovered statistically substantial downregulation of IL-1β gene expression levels in all groups after 1 week, with no statistically significant difference between the cryotherapy and control groups. In addition, the study found a statistically significant increase in IL-10 gene expression levels in all groups after 1 week. 21 Furthermore, most research using intracanal cryotherapy failed to record pain until the seventh day. 46 47 48

The results of this study revealed that age and gender had no statistically significant effect on the intensity of postoperative pain. Moreover, postoperative analgesic intake was noted in seven patients from the saline group, two patients from the cryotherapy group, and two patients from the occlusal reduction group; however, the difference was not statistically significant.

The results of the study were dependent on patients' self-reporting through the VAS. Since pain assessment is subjective, variations in individual perceptions and interpretations may impact the overall reliability of the study's findings, which arises as a limitation of this study. Moreover, the occurrence and severity of postoperative pain could be affected by various patient-related factors that were not explored in this study, such as anxiety and differences in pain threshold between individuals. Further research is recommended to investigate the influence of varying temperatures of saline on postoperative endodontic pain.

## Conclusion

Based on the results of the current study, we can conclude that cryotherapy is an effective and conservative method in decreasing the severity of postoperative pain following endodontic treatment of cases with symptomatic irreversible pulpitis and symptomatic apical periodontitis. Cryotherapy could be considered as effective as occlusal reduction, yet, more conservative in the management of postoperative endodontic pain.
