# Supplementary material for: Effect of Cryotherapy and Occlusal Reduction on Postoperative Endodontic Pain in Mandibular First Molars with Symptomatic Apical Periodontitis: A Prospective, Parallel, Double-Blinded Randomized Controlled Trial
Source: Eur J Dent. 2024 Nov 7;19(2):472–81. doi: 10.1055/s-0044-1791219 (PMC12020582; doi:10.1055/s-0044-1791219)
Supplement: Supplementary file 1 — Supplementary Material Appendix 1 [file 10-1055-s-0044-1791219-s2443484.pdf]

The Saline Group									
Patient #	Preop. PAIN	6HRS	24HRS	48HRS	72HRS	7DAYS	GENDER	AGE	MEDICATIONS
PT1	9	3	2	0	0	0	MALE	35	
PT2	2	0	0	0	0	0	MALE	29	
PT3	4	0	5	7	3	0	MALE	31	
PT4	3	0	0	0	0	0	FEMALE	45	
PT5	7	3	2	0	0	0	FEMALE	37	
PT6	9	4	3	0	0	0	FEMALE	40	
PT7	10	4	4	0	0	0	MALE	28	
PT8	7	0	0	0	0	0	FEMALE	37	
PT9	6	3	0	0	0	0	FEMALE	25	
PT10	7	3	2	0	0	0	MALE	34	
PT11	10	2	0	0	0	0	MALE	28	
PT12	10	6	6	4	1	0	MALE	26	X3 - 600mg ibuprofen at 6hrs and 24hrs.
PT13	9	7	3	1	0	0	FEMALE	48	X2 - 600mg ibuprofen at 6hrs and 24hrs.
PT14	4	2	2	1	1	0	MALE	30	
PT15	10	6	3	1	0	0	MALE	35	X1 - 600mg ibuprofen at 6hrs.
PT16	10	8	4	2	0	0	MALE	41	X2 - 600mg ibuprofen at 6hrs and 24hrs.
PT17	10	10	4	1	0	0	FEMALE	18	X1 - 600mg ibuprofen at 6hrs.
PT18	10	4	2	0	0	0	FEMALE	29	X1 - 600mg ibuprofen at 6hrs.
PT19	8	8	2	0	0	0	FEMALE	48	X1 - 600mg ibuprofen at 6hrs.
PT20	7	0	0	0	0	0	MALE	36	

The Cryotherapy Group									
Patient #	PreOP. Pain	6HRS	24HRS	48HRS	72HRS	7DAYS	GENDER	AGE	MEDICATIONS
PT1	9	2	2	3	2	0	MALE	26	
PT2	10	0	3	0	0	0	MALE	35	
PT3	9	4	0	0	0	0	MALE	29	

PT4	9	4	0	0	0	0	MALE	20	
PT5	10	6	2	0	0	0	MALE	25	X1 - 600mg ibuprofen at 6hrs.
PT6	10	2	0	0	0	0	MALE	38	
PT7	8	0	2	0	0	0	FEMALE	35	
PT8	6	4	0	0	0	0	MALE	26	
PT9	7	3	0	0	0	0	MALE	31	
PT10	4	0	0	0	0	0	MALE	23	
PT11	4	0	0	0	0	0	MALE	32	
PT12	6	0	0	0	0	0	MALE	29	
PT13	10	3	3	2	0	0	MALE	36	X2 - 600mg ibuprofen at 6hrs and 24hrs.
PT14	10	0	0	0	0	0	MALE	44	
PT15	5	2	0	0	0	0	MALE	32	
PT16	4	0	0	0	0	0	MALE	25	
PT17	7	0	0	0	0	0	MALE	39	
PT18	9	3	0	0	0	0	FEMALE	43	
PT19	8	2	0	0	0	0	MALE	33	
PT20	8	2	2	0	0	0	FEMALE	27	

The Occlusal Reduction Group									
Patient #	PreOP. Pain	6HRS	24HRS	48HRS	72HRS	7DAYS	GENDER	AGE	
PT1	9	0	0	0	0	0	FEMALE	20	
PT2	8	0	0	0	0	0	FEMALE	33	
PT3	6	2	1	0	0	0	FEMALE	18	
PT4	5	2	2	1	0	0	FEMALE	44	
PT5	10	3	3	0	0	0	MALE	26	
PT6	10	4	2	0	0	0	FEMALE	36	
PT7	4	0	0	0	0	0	MALE	34	
PT8	4	0	0	0	0	0	MALE	29	
PT9	10	7	3	2	1	0	MALE	35	X1 - 600mg ibuprofen at 6hrs.
PT10	8	4	2	2	0	0	MALE	22	
PT11	9	4	3	2	0	0	MALE	27	X3- 600mg ibuprofen at 6hrs, 24hrs, & 48hrs.
PT12	10	3	0	0	0	0	MALE	35	
PT13	8	3	3	0	0	0	FEMALE	45	

PT14	10	5	3	3	0	0	MALE	33	
PT15	10	2	0	0	0	0	MALE	51	
PT16	8	0	0	0	0	0	MALE	37	
PT17	7	0	0	0	0	0	FEMALE	43	
PT18	7	0	0	0	0	0	FEMALE	33	
PT19	7	0	0	0	0	0	MALE	23	
PT20	7	4	2	0	0	0	MALE	23	
